# Development of a Luciferase-Based In Vitro Assay to Evaluate the Efficacy of Anti-Cryptosporidial Drugs Against *Cryptosporidium parvum*

**DOI:** 10.3390/ph19040576

**Published:** 2026-04-03

**Authors:** Rie Kubota, Coh-ichi Nihei, Yoshifumi Nishikawa

**Affiliations:** 1 National Research Center for Protozoan Diseases, Obihiro University of Agriculture and Veterinary Medicine, Inada-cho, Obihiro 080-8555, Japan; kubot1121@obihiro.ac.jp; 2 Institute of Microbial Chemistry (BIKAKEN), 3-14-23 Kamiosaki, Tokyo 141-0021, Japan; conihei@bikaken.or.jp

**Keywords:** *Cryptosporidium parvum*, luciferase-based assay, anti-*Cryptosporidium* drug, kijimicin

## Abstract

**Background/Objectives**: *Cryptosporidium parvum* is a major causative agent of cryptosporidiosis; however, progress in anti-cryptosporidial drug discovery has been hindered by the lack of robust and reproducible in vitro evaluation systems. In this study, we developed and optimized a luciferase-based in vitro assay to quantitatively monitor *C. parvum* growth in HCT-8 cells. **Methods**: Key experimental parameters affecting infection efficiency were systematically examined, including sodium taurocholate treatment, timing of medium replacement, and serum concentration. **Results**: Sodium taurocholate significantly enhanced parasite infectivity, and removal of non-invaded parasites at 3 h post-infection (hpi) resulted in approximately 2-fold and 3.7-fold increase in luciferase activity at 24 and 48 hpi, respectively, compared with untreated controls. In contrast, removal at 24 hpi led to only an approximately 2.5-fold increase at 48 hpi, consistent with stage-dependent differences in parasite development. Morphological analyses confirmed parasite differentiation from trophozoites to meronts, followed by progression toward sexual stages. Using the optimized assay system, we evaluated several anticoccidial compounds and demonstrated potent in vitro activity of monensin and its structural analog kijimicin, whereas diclazuril and toltrazuril exhibited limited efficacy. **Conclusions**: Collectively, this luciferase-based platform provides a reliable and quantitative tool for anti-cryptosporidial drug screening and will facilitate future therapeutic development against *C. parvum*.

## 1. Introduction

*Cryptosporidium* is a protozoan parasite that colonizes the intestinal tract of a wide range of hosts, including both humans and animals. Infection with this parasite causes cryptosporidiosis, an acute diarrheal disease that can vary in severity from mild to life-threatening, particularly in young children and immunocompromised individuals, such as those with HIV/AIDS or those undergoing transplantation or chemotherapy. The global prevalence of *Cryptosporidium* infection in humans has been estimated at approximately 7.6%, with some countries reporting rates as high as 69.6% [1]. Moreover, a meta-analysis evaluating the long-term consequences of *Cryptosporidium*-associated diarrhea identified the parasite as the fifth leading cause of diarrheal disease in children under five years of age, contributing to an estimated 48,000 deaths and 4.2 million disability-adjusted life years annually [2].

Although an effective vaccine has been developed for use in cattle [3], no licensed vaccine is currently available for humans. Nitazoxanide remains the only approved therapeutic agent for cryptosporidiosis in immunocompetent individuals [4]. However, its efficacy is limited in immunocompromised adults, and even prolonged treatment regimens have demonstrated poor clinical outcomes in HIV-infected children under five years of age [5,6,7]. Cryptosporidiosis in animals also poses a substantial threat to the livestock industry, with infection-associated economic losses estimated to reach several million dollars worldwide [8,9]. Currently, halofuginone lactate (HFL), a synthetic quinazolinone derivative, is the approved therapeutic agent for the treatment of cryptosporidiosis in livestock [10,11]. However, treatment with HFL has certain limitations, and drug residues in the body are a concern [12,13,14]. In addition to HFL, other compounds such as paromomycin are also used in veterinary practice. While current control strategies are generally effective, the combination of these limitations highlights the urgent need for new, effective, and affordable anti-cryptosporidial drugs.

Leesombun et al. reported that kijimicin, a naturally occurring polyether ionophore, is a promising therapeutic candidate against *Toxoplasma gondii*, a zoonotic apicomplexan parasite [15]. Compared with the reference drug monensin, kijimicin exhibited reduced cytotoxicity toward host cells while maintaining potent selective activity against the parasite. In the present study, we employed HCT-8 cells to evaluate and compare the efficacy of nitazoxanide; the ionophore monensin and its structural analog kijimicin; and the anticoccidial agents diclazuril and toltrazuril against the asexual stages of *C. parvum*. The objective of this study was to develop and optimize a luciferase-based in vitro assay for quantitative evaluation of *C. parvum* growth and to assess its applicability for anti-cryptosporidial drug evaluation.

## 2. Results

### 2.1. Concentration-Dependent Luminescence of Luciferase-Expressing C. parvum Oocysts

**Figure 1:**
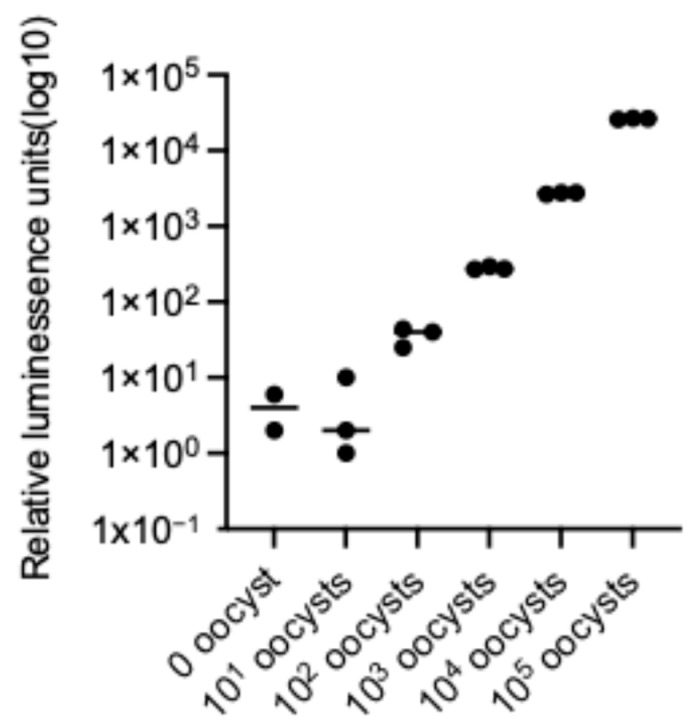
Linear correlation between oocyst number and luminescence intensity in luciferase-expressing *C. parvum*. Purified oocysts derived from the luciferase-expressing parasite were serially diluted (0–1 × 10^5^ oocysts), and luminescence was measured as relative luminescence units. Luminescence intensity increased proportionally with oocyst number within the tested range. Linear regression analysis revealed a strong positive correlation (r = 0.964).

Luminescence intensity increased proportionally with increasing oocyst number over a range of 0 to 1 × 10^5^ oocysts under drug-free conditions (Figure 1). Linear regression analysis demonstrated a strong positive correlation between oocyst number and luminescence signal (r = 0.964). Detectable luminescence signals were observed at oocyst concentrations of 1 × 10^2^ or higher.

### 2.2. Determination of Optimal In Vitro Conditions for Efficient Infection and Proliferation of C. parvum

**Figure 2:**
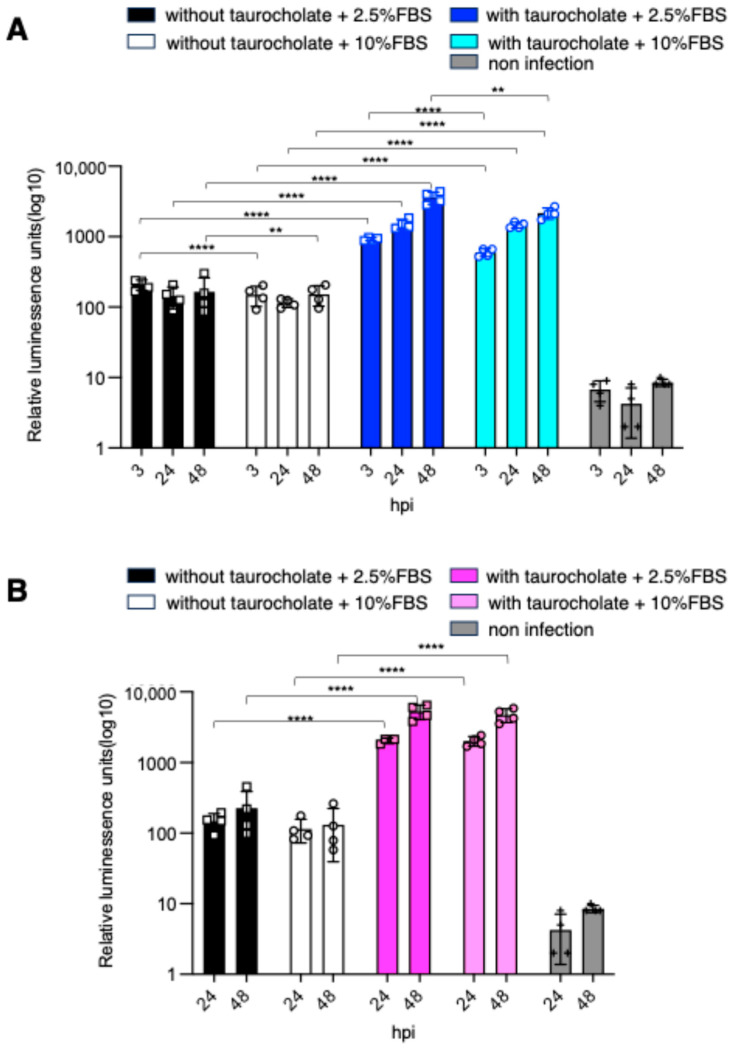
Optimization of in vitro conditions for efficient infection and proliferation of luciferase-expressing *C. parvum*. (**A**) Oocysts were treated with or without sodium taurocholate before infection. Non-invaded parasites were removed by medium replacement at 3 hpi, and luminescence was measured at the indicated time points. (**B**) Non-invaded parasites were removed at 24 hpi, and luminescence was measured at 24 and 48 hpi. Luminescence is presented as relative luminescence units. Non-infected controls are shown for comparison. Data represent the mean ± standard deviation of four technical replicates per condition, and the results are representative of two independent experiments with similar results. Statistical significance was determined by two-way ANOVA. **** *p* < 0.0001, ** *p* < 0.01. Only statistically significant differences are indicated.

Previous studies have reported various experimental conditions for the in vitro culture of *C. parvum* [16]. To establish optimal in vitro conditions for efficient infection and proliferation of *C. parvum*, we examined the effects of sodium taurocholate treatment, medium replacement timing, and fetal bovine serum (FBS) concentration. When oocysts treated with sodium taurocholate were used for infection and non-invaded parasites were removed by medium replacement at 3 hpi, luciferase activity increased to approximately 2-fold at 24 hpi and 3.7-fold at 48 hpi relative to the value at 3 hpi, in both 2.5% and 10% FBS conditions (Figure 2A). At 3 hpi, two-way ANOVA revealed significant main effects of sodium taurocholate treatment (*p* < 0.0001) and FBS concentration (*p* < 0.0001), as well as a significant interaction between these factors (*p* = 0.0003). At 24 hpi, a significant main effect of sodium taurocholate treatment was observed (*p* < 0.0001), whereas neither FBS concentration (*p* = 0.56) nor the interaction between the two factors (*p* = 0.86) was significant. At 48 hpi, significant main effects of sodium taurocholate treatment (*p* < 0.0001) and FBS concentration (*p* = 0.004) were observed, together with a significant interaction between the two factors (*p* = 0.004). When non-invaded parasites were removed at 24 hpi, sodium taurocholate treatment similarly enhanced parasite growth, resulting in approximately 2.5-fold higher luciferase activity at 48 hpi compared with untreated oocysts under both 2.5% and 10% FBS conditions (Figure 2B). At 24 hpi, a significant main effect of sodium taurocholate treatment was observed (*p* < 0.0001), whereas neither FBS concentration (*p* = 0.97) nor the interaction between the two factors (*p* = 0.74) was significant. At 48 hpi, a significant main effect of sodium taurocholate treatment was observed (*p* < 0.0001), whereas neither FBS concentration (*p* = 0.45) nor the interaction between the two factors (*p* = 0.65) was significant. Taken together, these results indicate that seeding host cells with sodium taurocholate-treated oocysts and removing non-invaded parasites at 3 hpi provides optimal conditions for efficient parasite invasion and subsequent in vitro proliferation.

### 2.3. Morphological Confirmation of C. parvum Developmental Stages

**Figure 3:**
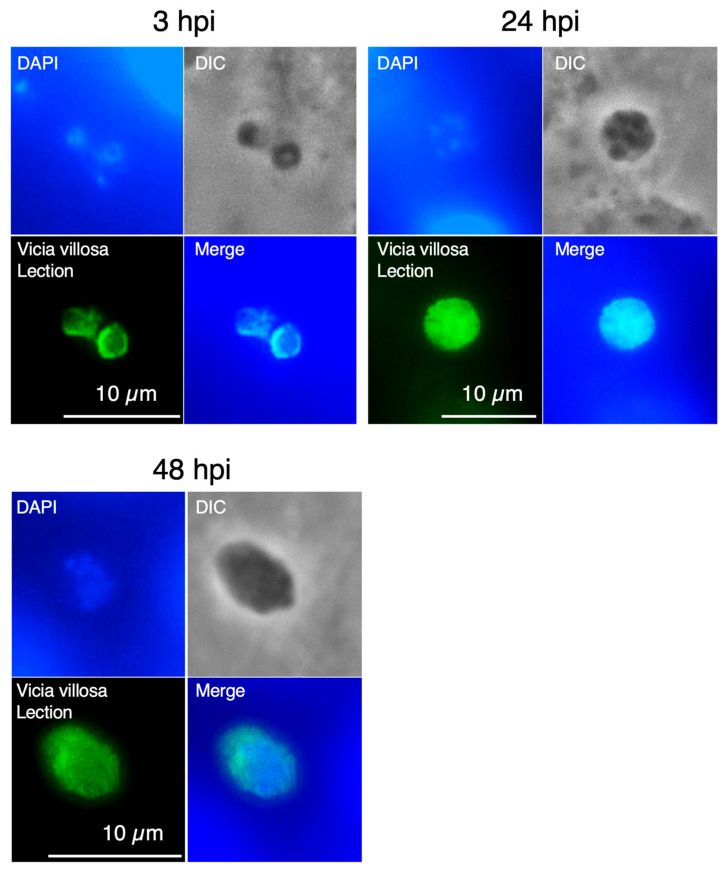
Morphological confirmation of stage-specific development of *Cryptosporidium parvum* in vitro. HCT-8 cells infected with luciferase-expressing *C. parvum* were analyzed by immunofluorescence assay at the indicated time points. Parasites were visualized using Vicia villosa lectin (VVL; green), and host cell nuclei were stained with Hoechst 33342 (blue). At 3 hpi, intracellular parasites containing a single nucleus were observed, consistent with trophozoites. At 24 hpi, parasites containing five or more nuclei were detected, consistent with developing meronts. At 48 hpi, multinucleated parasites were observed. Representative images from three independent experiments are shown. Scale bars, 10 µm.

The developmental progression of *C. parvum* in vitro has been described as follows: trophozoites are observed at 3 hpi, asexual replication advances by 24 hpi with the formation of meronts and merozoites, and sexual development proceeds by 48 hpi with the appearance of gametes and gamonts [17]. To confirm stage-specific differentiation under our culture conditions, morphological analyses were performed using immunofluorescence assays in HCT-8 cell cultures. At 3 hpi, intracellular parasites containing a single nucleus, consistent with trophozoites, were observed (Figure 3). At 24 hpi, parasites containing five or more nuclei were detected, consistent with developing meronts. Although we did not have antibodies specific for sexual-stage markers, multinucleated parasites were observed at 48 hpi. Based on nuclear morphology and developmental timing, these forms were considered consistent with gamont formation, suggesting progression to the sexual stage.

### 2.4. Monensin and Kijimicin Exhibit Potent Anti-Cryptosporidium Activity In Vitro

**Figure 4:**
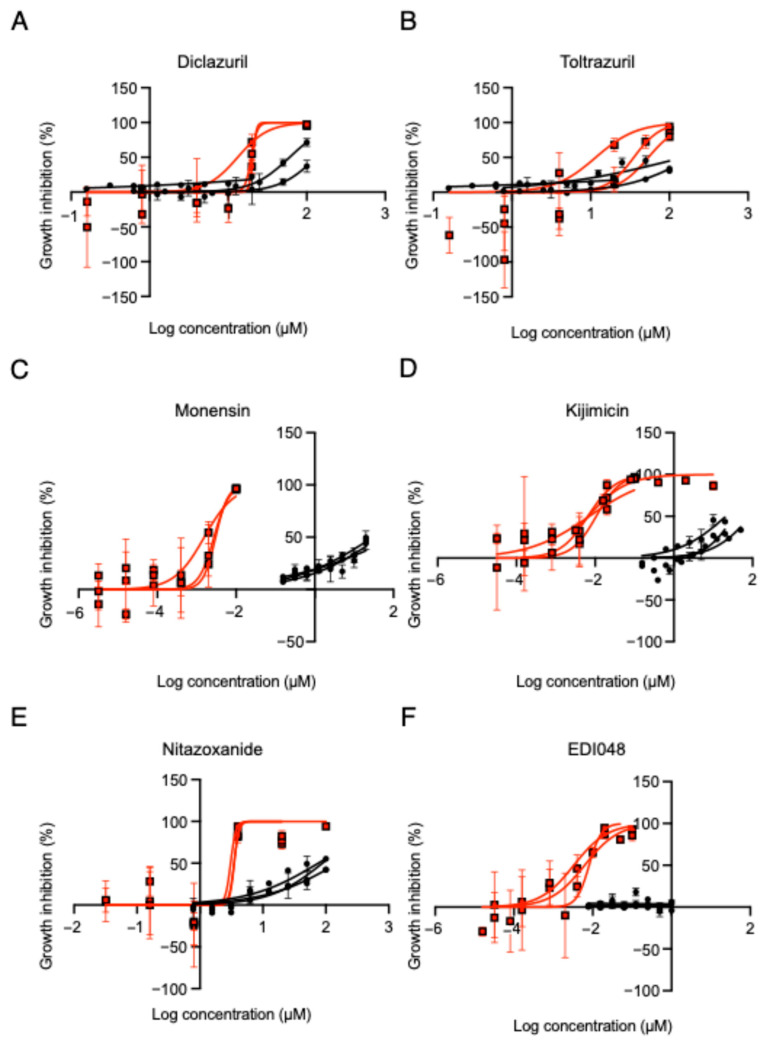
In vitro anti-*C. parvum* activity and cytotoxicity of candidate compounds. (**A**–**F**) Sigmoidal dose–response curves showing parasite growth inhibition (Red lines: half-maximal inhibitory concentration, IC_50_) and host cell cytotoxicity in HCT-8 cells (Black lines: half-maximal cytotoxic concentration, CC_50_) for each compound. Each curve represents one of three independent experiments, and the corresponding nonlinear regression fit is shown. IC_50_ and CC_50_ values were calculated by nonlinear regression analysis. Each independent experiment was performed in quadruplicate wells.

To evaluate the in vitro efficacy of four additional compounds against *C. parvum*, we determined their half-maximal inhibitory concentrations (IC_50_) for parasite growth and their cytotoxic concentrations (CC_50_) in HCT-8 host cells. Nitazoxanide, the standard anti-cryptosporidial drug, and EDI048, recently identified as a candidate anti-cryptosporidial compound exhibiting *Cryptosporidium* PI(4)K inhibitor activity [18], were included as reference controls. Diclazuril and toltrazuril exhibited IC_50_ values of 17,814 nM and 33,693 nM, respectively, which were substantially higher than that of nitazoxanide (IC_50_ = 3351 nM) (Table 1; Figure 4A,B,E). In contrast, monensin and kijimicin demonstrated markedly lower IC_50_ values of 2.4 nM and 7.7 nM, respectively, comparable to that of EDI048 (IC_50_ = 5.8 nM). IC_50_ values differed significantly among the compounds (one-way ANOVA, *p* < 0.0001). Tukey’s multiple comparison test revealed that toltrazuril exhibited significantly higher IC_50_ values than nitazoxanide, EDI048, monensin, and kijimicin (*p* < 0.01), whereas no significant differences were observed among the other compounds including diclazuril. CC_50_ values in HCT-8 cells are shown in Table 1.

## 3. Discussion

In the present study, sodium taurocholate treatment enhanced the infection efficiency of *C. parvum* compared to non-treatment with sodium taurocholate. In particular, when non-invaded parasites were removed at 3 hpi, luciferase activity at 24 and 48 hpi increased approximately 2-fold and 3.7-fold, as observed at 3 hpi. In contrast, when the medium was replaced at 24 hpi to remove non-invaded parasites, luciferase activity at 48 hpi increased only approximately 2.5-fold, as observed at 3 hpi. (Figure 2B). This difference may be attributable to stage-specific developmental dynamics of *C. parvum*. It is generally recognized that at 3 hpi, intracellular parasites are predominantly mononucleated trophozoites, whereas by 24 hpi, asexual replication progresses and multinucleated meronts are formed. By 48 hpi, parasites begin to transition to the sexual stage, with the appearance of gamonts [17]. When non-invaded parasites were removed at 3 hpi, only successfully invaded intracellular parasites remained, allowing efficient progression of subsequent asexual replication, which likely resulted in the marked increase in luciferase signal observed at 24 and 48 hpi. In contrast, when medium replacement was performed at 24 hpi, a proportion of parasites had likely already completed a round of asexual replication and produced merozoites, initiating a subsequent infection cycle. At this stage, parasite expansion would primarily originate from merozoites rather than from newly established trophozoites, potentially leading to different growth kinetics compared with infections initiated at the early trophozoite stage. Moreover, by 48 hpi, a subset of parasites may have transitioned to the sexual developmental stage. Because sexual differentiation represents a distinct biological process from asexual amplification, this shift may contribute to the relatively lower increase in luciferase signal. Taken together, the approximately 2.5-fold increase observed between 24 and 48 hpi in Figure 2B may be explained by (i) the shift in the proliferative starting population from trophozoites to merozoites and (ii) the transition from asexual replication to sexual development.

Although luciferase activity was used as a proxy for parasite growth, parasite development was also confirmed morphologically by immunofluorescence analysis. Nevertheless, further validation using independent quantitative methods such as quantitative PCR (qPCR) or microscopy-based parasite counting would strengthen the assay.

EDI048, a candidate anti-*Cryptosporidium* compound recently identified for its PI(4)K inhibitory activity [18], exhibited strong antiparasitic activity in this study, consistent with previous reports. Diclazuril, toltrazuril, and monensin, evaluated in this study, are well-established anticoccidial drugs that were primarily developed to target *Eimeria* species [19]. Although these compounds are thought to act on physiological processes conserved among apicomplexan parasites, our results demonstrate that drug susceptibility is not uniform across members of the phylum, even within Apicomplexa, and that *C. parvum* exhibits distinct sensitivity profiles. Notably, monensin and its structural analog kijimicin strongly inhibited *C. parvum* growth. Although diclazuril showed a relatively high IC_50_ value, no statistically significant difference was observed compared with the other compounds. These findings are consistent with previous reports indicating that monensin exhibits antiparasitic activity against *C. parvum*, whereas diclazuril shows little or no efficacy [20,21]. Kijimicin, a polyether ionophore structurally related to monensin, has been reported to possess anti-*Eimeria* and anti-*Toxoplasma* activities [15]. Polyether ionophores form lipid-soluble complexes with monovalent cations, particularly Na^+^, facilitating their transmembrane transport and thereby collapsing ionic gradients across biological membranes. In apicomplexan parasites, maintenance of ion homeostasis is critical for membrane potential regulation, osmotic stability, intracellular pH control, and calcium-dependent signaling pathways that are essential for invasion and intracellular replication [15,21,22]. Disruption of these processes can impair parasite development and viability. However, the molecular mechanisms regarding this activity remain unclear; the comparable activity of monensin and kijimicin supports the hypothesis that perturbation of ionic homeostasis contributes to their antiparasitic effects. In contrast, diclazuril and toltrazuril exhibited relatively weak activity against *C. parvum* despite their efficacy against other coccidian parasites. Diclazuril and toltrazuril are triazine derivatives that are thought to interfere with nuclear division and organelle development in coccidian parasites, including inhibition of apicoplast-associated functions [19]. *Cryptosporidium* lacks a canonical apicoplast and exhibits distinct intracellular biology compared with other apicomplexans. These differences may contribute to the reduced susceptibility of *C. parvum* to these compounds. These findings highlight the importance of parasite-specific drug evaluation and further support the utility of the present assay in distinguishing compound-specific activity against *C. parvum*. Overall, toltrazuril exhibited relatively reduced efficacy, whereas monensin and kijimicin showed strong antiparasitic effects. In addition, both compounds exhibited high selectivity indices. These findings indicate that monensin and kijimicin may represent promising candidate compounds for the treatment of cryptosporidiosis.

Furthermore, previous studies have suggested that in vitro antiparasitic activity does not necessarily translate into therapeutic efficacy in vivo. In particular, monensin has been reported to exhibit limited efficacy in in vivo models of cryptosporidiosis [20]. In the in vivo context, multiple factors—including pharmacokinetics, intestinal drug concentrations, and host immune responses—are likely to influence drug efficacy, imposing constraints distinct from those present in cell culture systems. Therefore, although the compounds identified in this study demonstrate promising in vitro activity, further validation using appropriate in vivo models is required to assess their therapeutic potential.

Several in vitro methods, such as high-content imaging and qPCR, have been developed to assess *Cryptosporidium* proliferation. High-content imaging allows for detailed morphological assessment of parasite development, but it is labor-intensive, requires specialized equipment and image analysis, and cannot reliably remove dead parasites. qPCR provides highly sensitive quantification of parasite DNA, but it does not directly reflect the live parasite burden and involves multiple processing steps [23]. In comparison with these approaches, the luciferase-based assay established in this study provides a rapid, simple, and quantitative method for evaluating parasite growth under defined conditions. This system enables efficient comparison of experimental parameters, such as excystation treatment and culture conditions, and facilitates the evaluation of compound efficacy. Therefore, the present assay represents a practical and scalable platform for anti-cryptosporidial drug screening.

## 4. Materials and Methods

### 4.1. Cell Culture and Parasites

HCT-8 (Human ileocecal adenocarcinoma cells, ATCC CCL-244) were cultured in Roswell Park Memorial Institute 1640 Medium (RPMI-1640; Sigma, St. Louis, MO, USA) supplemented with 10% fetal bovine serum (FBS; Biowest, Riverside, MO, USA), 100 U/mL penicillin-streptomycin (Fujifilm Wako, Osaka, Japan) at 37 °C under 5% CO_2_. Cells were passaged at approximately 70% confluence using 0.25% trypsin-EDTA (Gibco, Waltham, MA, USA). Cells were used for experiments between passage numbers 5 and 20. For immunofluorescence microscopy, cells were seeded inμ-slide 8-well high chambers (Watson, Tokyo, Japan). For drug screening, cells were seeded in white 96-well clear-bottom tissue culture plates (Corning Inc., Corning, NY, USA).

*Cryptosporidium parvum* Iowa nanoluciferase-expressing oocysts were a kind gift from Dr. Boris Striepen at the University of Pennsylvania [24]. *C. parvum* Iowa nanoluciferase-expressing oocysts that were less than one month old at the time of collection were used in all experiments.

### 4.2. Compounds

Nitazoxanide, the standard anti-cryptosporidial drug, and EDI048, recently identified as a candidate anti-cryptosporidial compound exhibiting *Cryptosporidium* PI(4)K inhibitor activity [18], were included as reference controls. Kijimicin, monensin, diclazuril and toltrazuril were anticoccidial drugs [15,19]. Kijimicin was provided by the Institute of Microbial Chemistry, Microbial Chemistry Research Foundation (BIKAKEN). Kijimicin was prepared in 100% ethanol at 10 mM. Monensin was obtained from Sigma and prepared in 100% ethanol at 20 mM. Nitazoxanide, Diclazuril and Toltrazuril were obtained from Tokyo Chemical Industry Co., Ltd., Tokyo, Japan and stored as stock solution in Dimethyl sulfoxide at 198 mM, 10 mM, and 36.4 mM, respectively. EDI048 was obtained from MedChemExpress, Monmouth Junction, NJ, USA and prepared in Dimethyl sulfoxide at 50 mM. All compounds were stored at  −30 °C until use. Working solutions were prepared in culture medium immediately before use. The tested compounds were selected based on their reported antiparasitic or anticoccidial activities. The range of drug concentrations was determined based on previous studies and preliminary experiments; the maximum drug concentration was set such that the DMSO content in the culture medium did not exceed 1%, and drug concentrations were evaluated using a four-fold serial dilution series [14,18].

### 4.3. Measure of Cryptosporidium parvum Iowa Nanoluciferase-Expressing Oocysts by NanoLuc Luciferase

To assess the relationship between oocyst number and luminescence intensity, purified luciferase-expressing *C. parvum* oocysts were serially diluted over a range of 0 to 1 × 10^5^ oocysts. Luminescence was measured using a luciferase assay and expressed as relative luminescence units. Linear regression analysis was performed to evaluate the correlation between oocyst number and luminescence signal. Each value represents the mean ± standard deviation of three technical replicates per condition, and the results are representative of two independent experiments with similar results.

### 4.4. 48 hpi Growth Inhibition Assay for Cryptosporidium parvum In Vitro IC_50_ Value Determination by NanoLuc Luciferase

HCT-8 cells were seeded into 96-well clear-bottom tissue culture plates at a density of 2.5 × 10^4^ cells per well and incubated overnight at 37 °C under 5% CO_2_. Oocysts were treated with 10 mM hydrochloric acid (10 min, 37 °C), followed by exposure to 2 mM sodium taurocholate (Tokyo Chemical Industry Co., Ltd., Japan) in PBS (+) (phosphate-buffered saline containing Ca^2+^ and Mg^2+^) (10 min, 16 °C) to initiate excystation [18]. The oocysts (2500 per well) were then added to the cells. At 3 hpi, the medium was washed with PBS twice and was replaced with serially diluted drug solutions at five different concentrations and parasite proliferation was assessed at 48 hpi at 37 °C under 5% CO_2_. Culture supernatant was removed from the wells, and 100 μL of Nano-Glo lysis buffer containing 1:50 of Nano-Glo substrate (Promega, Fitchburg, WI, USA) was added to the wells. The plate was incubated in the dark at room temperature for 3 min, and luminescence was measured using a SpectraMax iD5e reader (Molecular Devices, San Jose, CA, USA). Infection levels were quantified by measuring luciferase activity using a luminescence assay, which reflects parasite growth within host cells. Uninfected wells were included as negative controls, and positive control wells were without drug treatment with the parasite in all experiments. The IC_50_ values were calculated based on the detected fluorescence intensities and calculated by nonlinear regression using a four-parameter logistic model using GraphPad Prism 10 software. Briefly, their survival rates were plotted against the logarithm of drug concentration, and the curve fittings were performed by nonlinear regression to yield the drug concentration (IC_50_ values) that produced a 50% survival rate. Values were calculated by nonlinear regression analysis based on three independent experiments, each performed in quadruplicate wells. Data are presented as mean ± standard deviation of three independent experiments. Selectivity indices (SI) were determined as the ratio of CC_50_ to IC_50_.

### 4.5. Host Cell Cytotoxicity Assays

To determine the cytotoxicity of the extracts, cell viability assays were performed according to the published methodology [15]. Initially, a suspension of HCT-8 was prepared at a density of 2.5 × 10^5^ cells/well, and 100 μL of this suspension was dispensed into a 96-well plate. The cells were cultured for 24 h at 37 °C under 5% CO_2_. Test compounds were serially diluted in RPMI medium to obtain eight concentrations. The highest concentration corresponded to a 1:100 dilution of the stock solution, resulting in a final DMSO concentration of 1%. Compounds were added to the wells in quadruplicate and incubated for an additional 48 h. Vehicle control wells containing medium with 1% DMSO but no compound were included as negative controls. After incubation, the Cell Counting Kit-8 (CCK-8; Dojindo, Kumamoto, Japan) reagent was introduced to the wells, and the cells were incubated for a further 3 h at 37 °C under 5% CO_2_. The absorbance was subsequently measured at a wavelength of 450 nm. Cell viability was calculated according to the manufacturer’s instructions, and CC_50_ values were determined based on dose–response curves and calculated by nonlinear regression using a four-parameter logistic model in GraphPad Prism 10 software.

### 4.6. Isolation of Oocysts from Mouse Fecal

Fecal collections from the peak of infection were pooled, mixed with cold water, and stored at 4 °C for approximately one month. The fecal suspension was then mixed 1:1 with saturated sucrose and pelleted by centrifugation at 1000× *g* for 15 min. The supernatant was collected, washed with cold water, then centrifuged at 1000× *g* for 5 min to form a pellet. After filtration through a 100 μm mesh, the pellet containing oocysts was resuspended in PBS. Oocysts were separated using a cesium density gradient. Purified oocysts were washed with PBS and stored at 4 °C in penicillin-streptomycin-containing PBS for up to three months.

Interferon gamma knockout (*Ifnγ*^−/−^) mice were purchased from Jackson Laboratory. Mice of both sexes (5 females, 5 males, 20–25 g) were used for experiments, and mice were infected between the ages of 7 weeks and 8 weeks. Mice were individually housed (one mouse per cage). To maintain the parasite strain, Purified oocysts (1 × 10^5^) were administered orally, and fecals were collected daily for 30 days [25]. Mice were euthanized at 30 days post-infection under isoflurane anesthesia, followed by cervical dislocation. No treatment was administered after infection, and no mortality was observed during the experimental period.

### 4.7. Immunofluorescence Microscopy

For the immunofluorescence assay of extracellular sporozoites and oocysts, oocysts were excysted and placed on μ-slide 8-well high chambers. At the required time point, HCT8 cell monolayers were washed with PBS, fixed with 3% paraformaldehyde/PBS (Wako, Osaka, Japan) for 15 min, permeabilized with 0.25% Triton X-100 (Wako) for 10 min and blocked with 4% BSA/PBS (Wako) overnight at 4 °C. The antibodies were incubated for 1 h in 1% BSA/PBS along with the fluorescein labeled 1:2000 Vicia villosa lectin (VVL) (Vector Lab, Newark, CA, USA). Nuclei were stained by incubating with 1:10,000 Hoechst 33342 (Invitrogen, Waltham, MA, USA) in PBS for 10 min. Stained monolayers were washed with PBS, and the coverslips were mounted with ProLong Gold Antifade (Thermo Fisher Scientific, Waltham, MA, USA). The stained slides were examined under a ECLIPSE Ci-L plus microscope (Nikon, Tokyo, Japan). Uninfected wells were included as negative controls. Experiments were independently performed twice with similar results.

### 4.8. Statistical Analysis

Statistical analysis was performed using GraphPad Prism version 10 (GraphPad Software, San Diego, CA, USA). Data are presented as mean ± standard deviation (SD). Statistical analysis was performed using two-way ANOVA followed by Tukey’s multiple comparison test. Correlation analysis was performed using Pearson’s correlation coefficient (r) calculated in Microsoft Excel. IC_50_ values were compared using one-way ANOVA followed by Tukey’s multiple comparison test.

## 5. Conclusions

We developed and optimized a luciferase-based in vitro assay for quantitative evaluation of *C. parvum* growth. This platform enabled the assessment of parasite proliferation under defined culture conditions and identified monensin and kijimicin as compounds with potent in vitro activity. The assay provides a useful tool for anti-cryptosporidial drug evaluation, although further validation using additional methods and in vivo studies will be necessary.

## Figures and Tables

**Table 1:** In vitro anti-*Cryptosporidium parvum* activity and cytotoxicity of candidate compounds. Half-maximal inhibitory concentrations (IC_50_) for parasite growth and half-maximal cytotoxic concentrations (CC_50_) in HCT-8 host cells are shown for each compound. CC_50_ values were defined as the highest tested concentration when no cytotoxicity was observed within the tested range. Values with different letters indicate statistically significant differences (one-way ANOVA followed by Tukey’s multiple comparison test, *p* < 0.01).

Compound	Known Medicinal Effect	IC_50_ (nM) ±SD	CC_50_ (µM) ±SD	Selectivity Index
Diclazuril	Anti-*Coccidium*	17,814 ± 4016.84 ^ab^	86.84 ± 18.61	4.87
Toltrazuril	Anti-*Coccidium*	33,693 ± 21,301.83 ^b^	>100	>2.97
Monensin	Anti-*Coccidium*	2.44 ± 0.77 ^a^	>20	>8197
Kijimicin	Anti-*Toxoplasma*, anti-*Eimeria*	7.74 ± 1.96 ^a^	>20	>2585
Nitazoxanide	Anti-*Cryptosporidium*	3351 ± 244.77 ^a^	78.46 ± 19.81	23.41
EDI048	Anti-*Cryptosporidium*	5.88 ± 2.41 ^a^	>1	>170.12

## Data Availability

The original contributions presented in this study are included in the article. Further inquiries can be directed to the corresponding author.
